# Supporting parents in taking care of their infants admitted to a neonatal intensive care unit: a prospective cohort pilot study

**DOI:** 10.1186/s13052-017-0352-1

**Published:** 2017-04-17

**Authors:** Giuseppe De Bernardo, Maria Svelto, Maurizio Giordano, Desiree Sordino, Marina Riccitelli

**Affiliations:** 1 Department and Institution: Department of Emergency, NICU-A.O.R.N. Santobono-Pausilipon, Naples, Italy; 2 Department and Institution: Department of Molecular and Developmental Medicine, University of Siena, Siena, Italy

**Keywords:** Family care, Parental stress level, Newborn

## Abstract

**Background:**

Family-Centred Care (FCC) is recognized as an important component of all paediatric care, including neonatal care, although practical clinical guidelines to support this care model are still needed in Italy. The characteristics and services for families in Italian NICUs show a lack of organization and participation.

**Methods:**

The first aim was to compare satisfaction and stress levels in two groups of parents: an FCC group and a non-FCC group (NFCC). The second aim was to evaluate body weight gain in the newborns enrolled. This non-randomized, prospective cohort pilot study was conducted in a single level III NICU at a hospital in Naples, Italy. A cohort of newborns in the NICU, with their parents were enrolled between March 2014 and April 2015 and they were divided into two groups: the FCC group (enrolled between October 2014 and April 2015) remained in the NICU for 8 h a day with FCC model; the NFCC group (enrolled between March 2014 and September 2014) was granted access to the NICU for only 1 hour per day. At discharge, both parent groups completed the Parental Stressor Scale (PSS)-NICU and a questionnaire to assess their satisfaction. In addition, we compared scores from the mothers and fathers within and between groups and the body weights of the newborns in the two groups at 60 days.

**Results:**

Parents participating in the FCC group were more satisfied and less stressed than those in the NFCC group. Infants in the FCC group also showed increased body weight after 60 days of hospital stay.

**Conclusions:**

Despite our small population, we confirm that routine adoption of a procedure designed to apply a FCC model can contribute to improving satisfaction and distress among preterm infants’ parents. Future multi-centre, randomized, controlled trials are needed to confirm these findings.

## Background

The neonatal intensive care unit (NICU) environment is dramatically different from the maternal womb [1]. In the NICU, premature infants and their underdeveloped brains are exposed to negative sensory inputs, such as variations in temperature; touch; vestibular, gustatory, and olfactory sensations; noise; light; oxygen; and nutrients. In addition, premature newborns are separated physically, psychologically and emotionally from their parents [2]. All of these harsh conditions are a significant source of stress for premature infants and have a negative influence on their neurodevelopmental outcomes [3, 4]. Based on the previous work of Brazelton, in 1986, Als’ “Synactive Theory” [5] suggested that the neurodevelopmental subsystem interaction between the neonate’s internal functioning, the environment, and caregivers was the foundation of the neonatal developmental process: if a lack of equilibrium occurs within one subsystem, all other subsystems are affected [6]. Accordingly, Als introduced the concept of developmental care, a strategy to address the environmental issues of the NICU in order to reduce preterm infants’ stress, including control of external stimuli, centring nursery care procedures in time, and containing infants in a manner similar to what they experienced during the intrauterine period. Each preterm infant needs a specific combination of these concepts, as described in the ‘Newborn Individualized Developmental Care and Assessment Program’ (NIDCAP) [5]. Here, the family is the infant’s primary coregulator in order to reduce infant stress, and the caregivers must encourage greater parental involvement [7]. Based on this strategy, many programmes have been developed, such as kangaroo care, skin-to-skin care, and family-centred care (FCC) [2]. FCC is focused on the family’s role as the centre of the healthcare delivery system and on respect, communication, participation, collaboration and inclusion of the family in all aspects of their baby’s care [8]. The American Academy of Pediatrics has recognized the FCC concept as an important component of all paediatric care, including neonatal care, and has recommended that “conducting attending physician rounds (i.e., presentations and rounds discussions) in the patients’ rooms with the family present should be standard practice” [9, 10]. Davidson et al. indicated that when providing FCC, parents need to have the opportunity to participate in rounds so that they can ask questions and clarify information [11]. Unrestricted parental presence in the NICU, parental involvement in infant caregiving, and open communication with parents are the basic tenets of FCC [12] and several recent studies have demonstrated that higher-quality developmental care can improve pulmonary outcomes [1], reduce intraventricular haemorrhages [2], reduce the total length of the hospital stay [13], and improve neurobehavioral stability [4, 14]. The FCC model incorporates the family into the care of their baby, recognizing parents as fundamental members of the NICU team and as protagonists of their baby’s development [15]. This model requires special training for caregivers and families [16]. Parent education is essential to the success of the FCC programme. On first access, participants should communicate with the healthcare team, be properly prepared and become familiar with the NICU environment. FCC is based on principles of respect, communication and collaboration among healthcare workers and family members [10, 17, 18]. FCC has become a model of neonatal care worldwide [19], though its implementation sometimes presents difficulties [20–22]. Ashbaugh et al. [23] stated that practical guidelines are needed for applying already published NDICAP guidelines. Moreover, opening the NICU can be difficult to achieve. For instance, Greisen at al showed that unrestricted parental presence is not yet uniformly accepted among European NICUs. Parents are allowed access at any time in all units in Sweden, Denmark and the UK, in 90% of units in the Netherlands and Belgium, in 71% of units in France, but in only 30% of units in Italy and Spain [24]. Separation of parents from their baby in the NICU is usually related to parental depression, post-traumatic stress disorder, anxiety and other stress-related disorders [15]. With improvements in the care of the preterm infant, smaller and sicker infants are now surviving, yet having an infant hospitalized in the NICU remains a stressful experience for parents [25] in relation to several conditions, such as alterations in parenting roles, environment and staffing [14]. During the NICU stay, parents must cope with an unexpected birth and an altered pathway of parenthood [26]. Recently, Montirosso et al. [27] affirmed that mothers in NICUs undergo high levels of stress, mostly because of parental role marginalization. Many studies have focused on mothers, leaving out fathers, whereas Provenzi et al. found that fathers have a multi-dimensional emotional, cognitive and behavioural experience of preterm birth and NICU stay that is redefined across the NICU journey [28]. Indeed, fathers and mothers reported different parental experiences during the NICU stay. Mothers talk about their infant’s health status, and they affirm a deep sense of alienation, whereas fathers need information and practical support from nurses [26]. Comparing parental needs and perceptions is fundamental to improving developmental care in order to tailor specific support for every preterm newborn and his/her parents. The characteristics and services for families in Italian NICUs show a lack of organization and participation [29], and procedures are not enforced in each NICU in the same way or for an adequate amount of time. Moreover, there is no generally accepted definition of developmental care [30], and practical clinical guidelines to adequately support care interventions are still lacking in Italy. In addition, no study specifically addressing surgical neonatal diseases has applied FCC to the NICU context. Thus, it is at the discretion of individual intensive care units to promote FCC practices without a strong supporting evidence base.

## Methods

### Aims

Our primary aim was to compare satisfaction and stress levels between parents in an FCC group and a non-FCC (NFCC) group. Furthermore, we compared the satisfaction and stress levels of mothers and fathers within the FCC and NFCC groups and between the two groups. The secondary aim was to evaluate newborn body weight gain at 60 days after admission in the two groups.

### Participants

This non-randomized, prospective cohort pilot study was carried out from March 2014 to April 2015 in the NICU at the Santobono-Pausilipon Level III Hospital in Naples, Italy. The study was approved by the Ethics Committee of Santobono-Pausilipon Hospital, and written informed consent was obtained from the families. We recruited only Italian patients who lived in Campania. Eligibility criteria for newborns included patients who had recovered for at least 30 days from surgical diseases resolved with a single operation. The pathologies included oesophageal atresia, post-haemorrhagic hydrocephalus and diaphragmatic hernia. The exclusion criteria for participation consisted of twins, major complications and the absence of informed consent. The inclusion criteria for parents consisted of an age > 18 years, no single-parent families, no manifest psychiatric or cognitive pathologies and no drug addiction. Moreover, we selected only parents who lived in Naples.

### Enrolment and group composition

All eligible neonates and their parents were enrolled in the study. The NFCC group was enrolled from March 2014 to September 2014, and during this period, parents were permitted to visit their baby for 1 hour a day. The FCC group was enrolled from October 2014 to April 2015, and parents had access to their infants for up to 8 h a day during that time.

### Procedures

The implementation of the FCC model required a widening of the NICU. Two rooms and one kitchen were designated for families. Every room could host two parents and provided a place where they could rest and, if required, stay the night. The NICU could hold 10 incubators, and a filter zone was built to access the ward. During visiting hours, paediatric nurses taught parents the correct procedures and practices in the NICU in the interview room or in the ward for approximately 10 days. The NICU’s rules consisted of specified visiting hours, procedures to prevent the risk of contamination, procedures that parents could carry out with their infants, clinical bedside rounds hours, medical interview hours, and use of the rooms and kitchen. Both mothers and fathers could access the NICU from 10:00 to 18:00, but they had to wash their hands and don gowns in the filter zone before entering to reduce the risk of contamination. Initially, parents could only observe their infants and routine procedures such as venepuncture, heel stick and mechanical ventilation. When it was possible and if the parents felt ready, they participated with paediatric nurses in the care of their infants by bathing, changing diapers, breastfeeding and holding them during painful procedures. Parents could observe the clinical bedside rounds and hold meetings with physicians in the interview room from 15:00 to 16:30 and, if necessary, from 19:00 to 20:00. The physician explained to the parents the newborn’s clinical situation and tried to establish a therapeutic alliance with them. On the day of the patient’s discharge, the parents in both groups completed two questionnaires regarding their satisfaction and stress level with respect to their experience in the NICU. We asked the parents to complete the questionnaires separately to obtain answers from both mothers and fathers. It was not easy to get responses from both parents; therefore, one of the authors of the study called parents who were absent at the time of discharge.

### Instruments

During the length of stay of the newborns, one of the authors (M.S.) who was aware of the study aims recorded the following parameters in a database (Excel 2007) for evaluation of the samples’ homogeneity:

- Nationality, age and level of education of the parents

For newborns, the author recorded the following:

- Gestational age, Apgar score at 1 and 5 min, body weight at birth, body weight at admission to the NICU and at 60 days after admission, length of stay and pathologies

Paediatric nurses who were on duty measured the body weight of the newborns with a Seca BabyScale 354. Two questionnaires were used to assess the experiences of the parents. The first questionnaire was the satisfaction survey validated by Abdel-Latif ME et al. [20], which includes a total of 9 questions and measures three subscales of parents’ satisfaction with the healthcare team:

- Knowledge and Understanding (3 items) - satisfaction related to adequate and timely information about the baby’s condition
- Communication and Collaboration (3 items) - satisfaction with communication and collaboration with the healthcare team
- Privacy and Confidentiality (3 items) - satisfaction with respect of patient privacy

Parents are asked to rate each item on a five-point Likert scale from “strongly disagree (1)” to “strongly agree (5)”. The second questionnaire was the Parental Stressor Scale-NICU (PSS-NICU), a validated scale [17, 20, 22, 27] that measures how much stress parents have experienced because of their baby’s illness and hospitalization. This questionnaire included a total of 22 questions and measured the stress of three dimensions of parental experience during the NICU stay:

- Parental Role Alteration (8 items) - stress related to the alteration in the parental role because of the difficulty of taking care of their infants
- Infant Appearance (8 items) - stress related to infants’ appearance and behaviour
- Sight and Sound (6 items) - stress related to the NICU physical environment

Parents were asked to rate each item on a five-point Likert scale from “not stressful (1)” to “extremely stressful (5)”. An overall stress level score was computed for each subscale because the study’s focus was on the stress level of the parents. Parents who did not have the experience described in an item received a score of 1, and the median of each item was calculated [22]. The original version of the PSS-NICU was translated into Italian by the authors and revised by an English translator. The questionnaires were included in the analysis only if at least 90% of the items were completed.

### Statistical analysis

Analyses of the differences in clinical outcomes were performed by a statistician who was aware of the study aims using IBM SPSS Statistics for Windows, Version 20.0 (Armonk, NY: IBM Corp). Data with a normal distribution were analysed with the Kolmogorov-Smirnov test. Homogeneity of data groups was assessed by unpaired *t*-test or chi-square test if the data were, respectively, parametric or non-parametric. The Kruskal-Wallis (K-W) test was used to test the differences in the scores of the two questionnaires administered to parents of both groups. Considering that we obtained data from all mothers and fathers, the K-W test was performed to compare the scores of the mothers and fathers within each group. The same test was also used to compare the scores between the mothers and between fathers of the two groups. As a last step, newborn birth weight in both groups at 60 days was compared with an unpaired *t*-test. We considered *p* < 0.05 to be statistically significant.

## Results

**Fig. 1:**
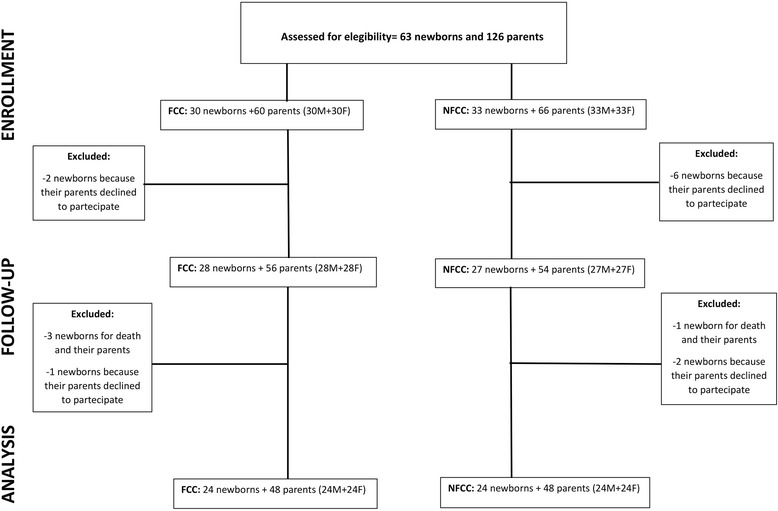
Flow diagram of the study population from assessment for eligibility to analysis

A total of 60 parents and 30 newborns in the FCC group and 66 parents and 33 newborns in the NFCC group were approached for recruitment. However, during hospitalization, 11 parents declined to participate and 4 newborns died, as reported in Fig. 1. In the final analysis, 96 parents (48 mothers and 48 fathers) were included with their newborns (48). The FCC group was composed of 24 mothers and 24 fathers and their 24 children, and the remaining 24 mothers and 24 fathers and their 24 children composed the NFCC group.

### Characteristics of neonates and parents at baseline

Preliminary analysis of the data validated the homogeneity of the samples. There were no differences between the characteristics of the children involved in FCC and those involved in NFCC. Furthermore, socio-demographic data on the parents were evaluated for homogeneity, but the analysis did not show any differences (Table 1).

**Table 1:** Baseline factors

		Total	FCC	NFCC	*P*-value
			Mean (±SD)	Mean (±SD)	
Parents	Nationality, Italian^a^	96	50%	50%	*p* > 0.05
	Age	96	34.81 (4.91)	35.65 (5.39)	*p* > 0.05
	Primary school^a^	96	4%	2%	*p* > 0.05
	Lower secondary school^a^		25%	33%	
	High school^a^		48%	48%	
	University degree^a^		23%	17%	
Newborns	Gestational age at birth, weeks	48	32.7 (5.25)	34.2 (5.25)	*p* > 0.05
	Apgar score, 1 min	48	5.41 (1.95)	6.37 (1.52)	*p* > 0.05
	Apgar score, 5 min	48	7.75 (0.67)	8.08 (0.88)	*p* > 0.05
	Birth weight, g	48	1,719.4 (865.3)	1,975.4 (732.4)	*p* > 0.05
	Body weight at admission, g	48	2,086.9 (887.6)	1,961.7 (758.7)	*p* > 0.05
	Oesophageal atresia^a^	48	46%	54%	*p* > 0.05
	Post-haemorrhagic hydrocephalus^a^		37%	29%	
	Diaphragmatic hernia^a^		17%	17%	
	Length of stay, days	48	86.58 (60.73)	89.71 (48.1)	*p* > 0.05

Sample characteristics at baseline. *N* = number; ^a^percentages are reported for nominal data

### Family survey data

The scores on the questionnaires and statistically significant differences are shown in Table 2. A comparison between the scores of mothers and fathers in both groups was performed to study the differences in NICU experiences, and the results are shown in Table 3. Furthermore, an additional comparison was performed between all mothers’ scores and between all fathers’ scores (Table 4). The item “When your baby can’t respond to you” was not answered by any parent, whereby it was assigned the score of 1. As a final step, we performed an analysis of newborn body weight at 60 days. The difference in body weight at 60 days between the cases in the FCC group and controls in the NFCC group was statistically significant (3,276.8 ± 1,016.8 g and 2,678.5 ± 628.8 g, respectively; *p* > 0.05).

**Table 2:** Evaluation of the families’ satisfaction and stress level

		FCC	NFCC	*p*_value
Knowledge and understanding		Median (5°–95°)	Median (5°–95°)	
I have received adequate information about my baby’s condition and management.		5 (3,45–5)	4 (3–5)	<0,05
The health care team explained things thoroughly using easy to understand language.		5 (4–5)	4 (3,45–5)	<0,05
The information I have received has been appropriate and timely.		5 (3,45–5)	3 (2–4)	<0,05
Communication and collaboration				
In the last week I have been able to communicate effectively with my baby’s health care team.		5 (4–5)	4 (3–5)	<0,05
In the last week I have collaborated with my baby’s health care team in the planning of care for my baby.		5 (4–5)	1 (1–2)	<0,05
In the last week I have been able to ask the health care team questions about my baby’s care.		5 (4–5)	4 (3–5)	>0,05
Privacy and confidentiality				
In the last week the privacy of my baby’s care was always considered and upheld		5 (4–5)	5 (3,45–5)	<0,05
In the last week the confidentiality of my baby’s care was always considered and upheld		5 (4–5)	5 (3,45–5)	<0,05
In the last week I have overheard information about other babies		1 (1–2)	1 (1–3)	>0,05
PSS: NICU				
Parental role alteration	Being separated from your baby	5 (4–5)	5 (4–5)	<0,05
	Not being able to regularly care for your baby (e.g., feed, nappy, hold)	4 (2–4,55)	5 (4–5)	<0,05
	Not having a chance to be alone with your baby	3 (1–4)	5 (3,45–5)	<0,05
	Not being able to share your baby with family and friends	4 (2–4,55)	5 (3–5)	<0,05
	Not being able to protect your baby from pain and painful procedures	5 (4–5)	5 (4–5)	>0,05
	Not being able to comfort or help your baby	5 (4–5)	5 (4–5)	>0,05
	The nurses and other staff seeming closer to the baby than you are	2 (1–3)	4 (2,45–5)	<0,05
	Not being able to hold your baby	4 (2–4,55)	5 (3–5)	<0,05
Infant appearance	Seeing your baby with tubes or IV lines in him/her	4 (2,45–5)	5 (4–5)	<0,05
	Seeing your child in pain	5 (4–5)	5 (4–5)	>0,05
	Having your child look afraid, be upset or cry a lot	5 (3–5)	5 (4–5)	<0,05
	Seeing your baby look sad	4 (3,45–5)	5 (4–5)	<0,05
	Seeing a needle or tube put in your baby	4 (2,45–5)	5 (4–5)	<0,05
	Seeing your baby have problems breathing	4 (1–5)	5 (4–5)	<0,05
	Seeing your baby surrounded by machinery and having medical treatments	3 (1–4)	5 (4–5)	<0,05
	When your baby can’t respond to you	1	1	>0,05
Sight and sound	Monitors and equipment in the room	3 (2–4)	5 (2,45–5)	<0,05
	The sudden sound of monitor alarms	3 (1–4)	4 (1,45–4,55)	<0,05
	The other sick children in the room	3 (1–3)	3 (1–3)	<0,05
	The large number of nurses, doctors, and other staff who work with your child	1 (1,45–4)	1 (1–5)	>0,05
	When other children in the hospital have a crisis	4 (1–3,55)	4 (1–4)	>0,05
	The needs of other parents in the hospital	1 (1–3,55)	2 (1–4)	<0,05

The evaluation of the parents’ satisfaction was measured by 3 sections: knowledge and understanding, communication and collaboration, privacy and confidentially. The evaluation of the parents’ stress level was measured by PSS: NICU

**Table 3:** Evaluation of the satisfaction and stress level between mothers and fathers

		FCC			NFCC		
		Median (5°–95°)		p_value	Median (5°–95°)		*p*_value
		Mother	Father		Mother	Father	
Knowledge and understanding							
I have received adequate information about my baby’s condition and management.		5 (3–5)	5 (3–5)	*p* > 0,05	4 (3–5)	4 (3–5)	*p* > 0,05
The health care team explained things thoroughly using easy to understand language.		5 (4–5)	5 (4–5)	*p* > 0,05	4 (3,25–5)	4 (3,25–5)	*p* > 0,05
The information I have received has been appropriate and timely.		5 (3–5)	5 (3–5)	*p* > 0,05	3 (2–4)	3 (2–4)	*p* > 0,05
Communication and collaboration							
In the last week I have been able to communicate effectively with my baby’s health care team.		5 (4–5)	5 (3,25–5)	*p* > 0,05	4 (3–5)	4 (3–5)	*p* > 0,05
In the last week I have collaborated with my baby’s health care team in the planning of care for my baby.		5 (4–5)	5 (4–5)	*p* > 0,05	1 (1–2)	1 (1–2)	*p* > 0,05
In the last week I have been able to ask the health care team questions about my baby’s care.		5 (4–5)	5 (4–5)	*p* > 0,05	4 (3–5)	4 (3–5)	*p* > 0,05
Privacy and confidentiality							
In the last week the privacy of my baby’s care was always considered and upheld		5 (4–5)	5 (4–5)	*p* > 0,05	4,5 (3,25–5)	5 (3,25–5)	*p* > 0,05
In the last week the confidentiality of my baby’s care was always considered and upheld		5 (4–5)	5 (4–5)	*p* > 0,05	5 (3,25–5)	4,5 (3,25–5)	*p* > 0,05
In the last week I have overheard information about other babies		1 (1–2)	1 (1–2)	*p* > 0,05	1 (1–4,5)	1 (1–3)	*p* > 0,05
PSS: NICU							
Parental role alteration	Being separated from your baby	5 (4–5)	5 (4–5)	*p* > 0,05	5	5 (4–5)	*p* < 0,05
	Not being able to regularly care for your baby (e.g., feed, nappy, hold)	4 (2–4,75)	4 (2–4,75)	*p* > 0,05	5	5 (3,25–5)	*p* < 0,05
	Not having a chance to be alone with your baby	3 (1–4)	3 (1–4)	*p* > 0,05	5 (3,25–5)	5 (3,25–5)	*p* > 0,05
	Not being able to share your baby with family and friends	4 (2–4,75)	4 (2–4,75)	*p* > 0,05	5 (3–5)	4,5 (3–5)	*p* > 0,05
	Not being able to protect your baby from pain and painful procedures	5 (4–5)	5 (4–5)	*p* > 0,05	5 (4–5)	5 (4–5)	*p* > 0,05
	Not being able to comfort or help your baby	5 (4–5)	5 (4–5)	*p* > 0,05	5 (4–5)	5 (4–5)	*p* > 0,05
	The nurses and other staff seeming closer to the baby than you are	1,5 (1–3)	1,5 (1–3)	*p* > 0,05	4 (2,25–5)	4 (2,25–5)	*p* > 0,05
	Not being able to hold your baby	4 (2–4,75)	4 (2–4,75)	*p* > 0,05	5 (3,25–5)	5 (3–5)	*p* > 0,05
Infant appearance	Seeing your baby with tubes or IV lines in him/her	4 (2,25–5)	4 (2,25–5)	*p* > 0,05	5 (4–5)	5 (4–5)	*p* < 0,05
	Seeing your child in pain	5 (4–5)	5 (4–5)	*p* > 0,05	5 (4–5)	5 (4–5)	*p* > 0,05
	Having your child look afraid, be upset or cry a lot	5 (3–5)	5 (3–5)	*p* > 0,05	5 (4–5)	5 (3,25–5)	*p* > 0,05
	Seeing your baby look sad	4 (4–5)	4 (3–5)	*p* > 0,05	5 (4–5)	5 (3,25–5)	*p* < 0,05
	Seeing a needle or tube put in your baby	4 (2,25–5)	4 (2,25–5)	*p* > 0,05	5 (4–5)	5 (4–5)	*p* < 0,05
	Seeing your baby have problems breathing	4,5 (3,25–5)	4 (3–5)	*p* > 0,05	5 (4–5)	5 (3,25–5)	*p* < 0,05
	Seeing your baby surrounded by machinery and having medical treatments	3 (1–5)	3 (1–5)	*p* > 0,05	5 (4–5)	5 (4–5)	*p* > 0,05
	When your baby can’t respond to you	1	1	*p* > 0,05	1	1	*p* > 0,05
Sight and sound	Monitors and equipment in the room	3 (1–4)	3 (1–4)	*p* > 0,05	5 (4–5)	5 (4–5)	*p* > 0,05
	The sudden sound of monitor alarms	4 (2–4)	3 (2–4)	*p* > 0,05	4 (2,25–5)	4 (2,25–5)	*p* > 0,05
	The other sick children in the room	3 (1–4)	2,5 (1–4)	*p* > 0,05	3 (1,25–4,75)	3 (1,2–4,75)	*p* > 0,05
	The large number of nurses, doctors, and other staff who work with your child	1 (1–3)	1 (1–3)	*p* > 0,05	1 (1–3)	1 (1–3)	*p* > 0,05
	When other children in the hospital have a crisis	4 (1,25–4)	3 (1,25–4)	*p* > 0,05	4 (1,5–5)	4 (1–5)	*p* > 0,05
	The needs of other parents in the hospital	1 (1–4)	1 (1–3)	*p* > 0,05	2 (1–4)	2 (1–4)	*p* > 0,05

The evaluation of the satisfaction between mothers and fathers was measured by 3 sections: knowledge and understanding, communication and collaboration, privacy and confidentially. The evaluation of the stress level between mothers and fathers was measured by PSS: NICU

**Table 4:** Evaluation of the satisfaction and stress level between mothers and between fathers

		M-FCC	M-NFCC	*p*_value	F-FCC	F-NFCC	*p*_value
		Median (5°–95°)			Median (5°–95°)		
Knowledge and understanding							
I have received adequate information about my baby’s condition and management.		5 (3–5)	4 (3–5)	*p* < 0,05	5 (3–5)	4 (3–5)	*p* < 0,05
The health care team explained things thoroughly using easy to understand language.		5 (4–5)	4 (3,25–5)	*p* < 0,05	5 (4–5)	4 (3,25–5)	*p* < 0,05
The information I have received has been appropriate and timely.		5 (3–5)	3 (2–4)	*p* < 0,05	5 (3–5)	3 (2–4)	*p* < 0,05
Communication and collaboration							
In the last week I have been able to communicate effectively with my baby’s health care team.		5 (4–5)	4 (3–5)	*p* < 0,05	5 (3,25–5)	4 (3–5)	*p* < 0,05
In the last week I have collaborated with my baby’s health care team in the planning of care for my baby.		5 (4–5)	1 (1–2)	*p* < 0,05	5 (4–5)	1 (1–2)	*p* < 0,05
In the last week I have been able to ask the health care team questions about my baby’s care.		5 (4–5)	4 (3–5)	*p* < 0,05	5 (4–5)	4 (3–5)	*p* < 0,05
Privacy and confidentiality							
In the last week the privacy of my baby’s care was always considered and upheld		5 (4–5)	4,5 (3,25–5)	*p* < 0,05	5 (4–5)	5 (3,25–5)	*p* > 0,05
In the last week the confidentiality of my baby’s care was always considered and upheld		5 (4–5)	5 (3,25–5)	*p* < 0,05	5 (4–5)	4,5 (3,25–5)	*p* < 0,05
In the last week I have overheard information about other babies		1 (1–2)	1 (1–4,5)	*p* > 0,05	1 (1–2)	1 (1–3)	*p* > 0,05
PSS: NICU							
Parental role alteration	Being separated from your baby	5 (4–5)	5	*p* < 0,05	5 (4–5)	5 (4–5)	*p* > 0,05
	Not being able to regularly care for your baby (e.g., feed, nappy, hold)	4 (2–4,75)	5	*p* < 0,05	4 (2–4,75)	5 (3,25–5)	*p* < 0,05
	Not having a chance to be alone with your baby	3 (1–4)	5 (3,25–5)	*p* < 0,05	3 (1–4)	5 (3,25–5)	*p* < 0,05
	Not being able to share your baby with family and friends	4 (2–4,75)	5 (3–5)	*p* < 0,05	4 (2–4,75)	4,5 (3–5)	*p* < 0,05
	Not being able to protect your baby from pain and painful procedures	5 (4–5)	5 (4–5)	*p* > 0,05	5 (4–5)	5 (4–5)	*p* > 0,05
	Not being able to comfort or help your baby	5 (4–5)	5 (4–5)	*p* > 0,05	5 (4–5)	5 (4–5)	*p* > 0,05
	The nurses and other staff seeming closer to the baby than you are	1,5 (1–3)	4 (2,25–5)	*p* < 0,05	1,5 (1–3)	4 (2,25–5)	*p* < 0,05
	Not being able to hold your baby	4 (2–4,75)	5 (3,25–5)	*p* < 0,05	4 (2–4,75)	5 (3–5)	*p* < 0,05
Infant appearance	Seeing your baby with tubes or IV lines in him/her	4 (2,25–5)	5 (4–5)	*p* < 0,05	4 (2,25–5)	5 (4–5)	*p* < 0,05
	Seeing your child in pain	5 (4–5)	5 (4–5)	*p* > 0,05	5 (4–5)	5 (4–5)	*p* > 0,05
	Having your child look afraid, be upset or cry a lot	5 (3–5)	5 (4–5)	*p* < 0,05	5 (3–5)	5 (3,25–5)	*p* > 0,05
	Seeing your baby look sad	4 (4–5)	5 (4–5)	*p* < 0,05	4 (3–5)	5 (3,25–5)	*p* < 0,05
	Seeing a needle or tube put in your baby	4 (2,25–5)	5 (4–5)	*p* < 0,05	4 (2,25–5)	5 (4–5)	*p* < 0,05
	Seeing your baby have problems breathing	4,5 (3,25–5)	5 (4–5)	*p* < 0,05	4 (3–5)	5 (3,25–5)	*p* > 0,05
	Seeing your baby surrounded by machinery and having medical treatments	3 (1–5)	5 (4–5)	*p* < 0,05	3 (1–5)	5 (4–5)	*p* < 0,05
	When your baby can’t respond to you	1	1	*p* > 0,05	1	1	*p* > 0,05
Sight and sound	Monitors and equipment in the room	3 (1–4)	5 (4–5)	*p* < 0,05	3 (1–4)	5 (4–5)	*p* < 0,05
	The sudden sound of monitor alarms	4 (2–4)	4 (2,25–5)	*p* > 0,05	3 (2–4)	4 (2,25–5)	*p* < 0,05
	The other sick children in the room	3 (1–4)	3 (1,25–4,75)	*p* > 0,05	2,5 (1–4)	3 (1,2–4,75)	*p* < 0,05
	The large number of nurses, doctors, and other staff who work with your child	1 (1–3)	1 (1–3)	*p* > 0,05	1 (1–3)	1 (1–3)	*p* > 0,05
	When other children in the hospital have a crisis	4 (1,25–4)	4 (1,5–5)	*p* > 0,05	3 (1,25–4)	4 (1–5)	*p* > 0,05
	The needs of other parents in the hospital	1 (1–4)	2 (1–4)	*p* > 0,05	1 (1–3)	2 (1–4)	*p* > 0,05

The evaluation of the satisfaction was measured by 3 sections: knowledge and understanding, communication and collaboration, privacy and confidentially. The evaluation of the stress level was measured by PSS: NICU

### Limitations of the study

The present study is not without limitations. First, the small sample size limited the detection of differences between the two groups. This small sample was derived from a selected population of preterm newborns with surgical disease and their parents. Moreover, our NICU received infants from all of Campania’s cities, some of which are located far from our hospital, and we excluded these patients because of the increased level of stress of being far from home. As mentioned in previous studies [20], conducting a double-blind study in the NICU is not possible because of the nature of the intervention. Moreover, it was not possible to achieve randomization because of the timed mode of enrolment. In addition, the socio-economic status of families was not calculated with the Hollingshead classifications [27]. The scores of mothers and fathers were not controlled for the source of stress (e.g., socio-economic status), which may be a reason for the variation in the scores. Last, we were not able to distinguish which procedures were the major sources of stress for the parents of preterm newborns.

## Discussion

The primary aim of this pilot study was to evaluate differences in satisfaction and stress level between parents in the FCC group, who had access to the NICU for 8 h a day, and parents in the NFCC group, who had access for only 1 h per day. The programme was adapted to our hospital and allowed families to participate in newborn care. The plan was to expand parental presence in the NICU and have the parents become an integral part of their newborn’s care, with the objective of opening the department 24 h a day to match other care-by-parent models. After attending training, parents in the FCC group were able to join as active members under the supervision of health professionals and cooperate with staff by caring for their children in the NICU [21, 24, 31]. As we expected, the comparison between the two groups revealed greater satisfaction among parents in the FCC group (Table 2). The information that the parents received was appropriate and timely; they were able to communicate effectively with the healthcare team and felt that their privacy had been considered and respected. These results are in accordance with previous literature supporting FCC as an important quality standard improvement to developmental care [8–10], and a method for promoting better exchange of information between clinicians and families [20]. Notably, however, both parent groups were satisfied regarding communication: no statistically significant difference was found between the FCC and NFCC groups with regard to specifically asking questions about the infant’s care. This finding could be related to a good quality time dedicated to communication in our model of developmental care but any way duration of communication needs to be improved. A policy of unlimited, open access for parents should ensure around-the-clock information and access to their baby [15]. An analysis of the PSS-NICU results revealed a statistically significant difference in stress between the two groups of parents (Table 2): those in the NFCC group felt more stressed than those in the FCC group. This alteration in the parental role resulted in lower scores in the FCC group than in the NFCC group, as those in the NFCC group felt they were not able to protect their child from painful procedures and were not able to comfort or help. In fact, several studies have shown that parental support during the NICU stay can reduce the parents’ own stress level [2, 20, 21]. The infant’s appearance was a source of stress for the parents of both groups, particularly those in the NFCC group, especially when they saw their baby in pain, supporting the notion that infant pain is strongly distressing for parents, especially for mothers. Montirosso affirmed [27] that this distress leads to frequent misperceptions of their baby’s behavioural cues, even labelling their babies as ‘difficult’ [15]. The surrounding environment and medical treatments were more stressful to parents in the NFCC group, possibly because these parents were not able to spend as much time in the NICU environment and were perceived as extraneous [6]. Our analysis showed that, in both groups, caregivers were not perceived as a source of stress. These data are in line with previous data on satisfaction, and both findings are likely related to the high level and appropriate quality of care provided to preterm infant families in both groups.

The parents of premature babies often lack support and opportunities to engage in parenting. Thus, in order to investigate differences in satisfaction and stress perception between mothers and fathers, we compared the questionnaire results among parents in each group (Table 3). Mothers and fathers in the FCC group perceived their NICU experience as satisfying and stressful in the same way with no statistically significant differences on any items. This finding could be related to the equal involvement of both parents in developmental care [8, 19], and it validates the hypothesis that FCC promotes both parental roles equally [2, 9–11, 19, 32]. Moreover, these data, according to Provenzi [26], reveal that the experience of becoming a parent of a preterm infant is very stressful because of the adjustment to the preterm birth and parental role adjustments. It is likely that FCC care for only 8 h is not sufficient to offset this stress. Additionally, in the NFCC group, the differences between the experiences of mothers and fathers were not large, although fathers appeared more stressed when the baby was alone, in pain, or subjected to painful procedures when they were not able to provide care. Additionally, Table 4 shows that fathers were more stressed by the separation from and suffering of their baby. This finding is in agreement with Provenzi et al. [28], who found that NICU fathers of preterm infants needed different and specific nursing support and interventions to sustain caregiving engagement and the transition to parenthood. This specific type of training was not possible in the NFCC group.

As observed in Table 4, the comparison between mothers of both groups and fathers of both groups showed us that mothers and fathers in the FCC group were more satisfied with the information they received, their communication with the health care team and the respect for privacy they encountered in the NICU compared to the parents in the NFCC group. Our data confirmed that mothers and fathers in the FCC group were less stressed than those in the NFCC group, with a reduction in parental role alteration and reduced impact of infant appearance. The comparison between mothers in the FCC and NFCC groups revealed that there was not a statistically significant difference in the perception of the NICU environment in the NFCC group. These findings do not differ from those reported in recent literature strongly promoting FCC [1, 5, 9]. Moreover, our findings support the implementation of FCC in our department, showing that parents need to be involved in their infant’s care to reduce both the infant’s stress and their own.

As shown in Table 4, items such as “not being able to protect your baby from pain and painful procedures” and “not being able to comfort or help your baby” were identified as sources of stress for both mothers and fathers in the two groups, supporting the hypothesis that FCC for only 8 h was not sufficient.

Finally, it is interesting to note that infants in the FCC group had a greater body weight than those in the NFCC group 60 days after the beginning of the study. Considering weight gain as a good health indicator, this finding could suggest, as shown in other literature reports [1], that an improvement in developmental care is a fundamental part of healthy development. Italian NICUs are not homogeneous, and FCC model are not universally adopted because the benefits to the NICUs are rarely investigated in terms of parent satisfaction and stress reduction [14, 24, 29]. We sought to contribute to improving the Italian NICU context in terms of organization and parental satisfaction. The plan was to expand parents’ presence in the NICU and have them became an integral part of their newborn’s care, with the objective of keeping the department open 24 h a day to match other care-by-parent models. After attending training, parents in the FCC group could join as active members to care for their children under the supervision of paediatric nurses [21, 24, 33]. There are many studies of FCC in the NICU, and many programmes have been investigated, such as creating opportunities for parent empowerment, neonatal individualized developmental care and assessment programmes, mother-infant transaction programmes, nursing child assessment teaching scales, kangaroo care, baby massage, and parental presence at clinical bedside rounds [10, 32, 34–38]. Abdel-Latif et al. [20] found that the inclusion of parents in bedside medical rounds as a part of FCC was strongly supported by parents who were satisfied and without increased stress levels. Jacobowski et al.[18] reported that NICU patient and family satisfaction and comprehension of the course of critical illness were recognized as relevant measures for the outcomes and benchmarks of high-quality NICU care. The application of this new model of care is not easy because it requires a new way of working with patients and families. However, O’Brien et al. [2] reported significantly decreased stress scores among parents involved in family-integrated care. The parents also found this approach to be very beneficial, and it enabled them to have greater confidence in their parenting skills at the time of discharge from the hospital.

## Conclusions

Our findings show that routine adoption of a procedure designed to apply a care-oriented model can contribute to improving satisfaction and distress among preterm infants’ parents. The FCC group showed greater satisfaction with how information was received in a timely and appropriate manner, while both groups were able to communicate with healthcare workers. The parents also felt that their privacy was considered and respected by the caregivers owing to the private rooms made available to them to discuss diagnoses and therapies. The level of stress was lower in the FCC group than in the NFCC group, although the stress level was still high in both groups. Thus, the quality and the duration of FCC must be improved in our department and in all Italian NICUs.

The obtained results show that the model of family integration has many advantages, although future multi-centre, randomized, controlled trials are needed to confirm these findings, due to spread FCC models in the Italian context, drawing up and introducing standard practical guidelines.

## Abbreviations

FCC: Family-centred care
NFCC: Non-family-centred care group
NICU: Neonatal intensive care unit
PSS: Parental stressor scale
